# Neural functional architecture and modulation during decision making under uncertainty in individuals with generalized anxiety disorder

**DOI:** 10.1002/brb3.1015

**Published:** 2018-06-21

**Authors:** Michal Assaf, Liron Rabany, Luis Zertuche, Laura Bragdon, David Tolin, John Goethe, Gretchen Diefenbach

**Affiliations:** ^1^ Olin Neuropsychiatry Research Center Institute of Living Hartford Hospital Hartford Connecticut; ^2^ Department of Psychiatry Yale School of Medicine New Haven Connecticut; ^3^ Psychology Department Binghamton University Binghamton New York; ^4^ Anxiety Disorders Center Institute of Living Hartford Hospital Hartford Connecticut; ^5^ Burlingame Center Institute of Living Hartford Hospital Hartford Connecticut

**Keywords:** ACC, amygdala, functional connectivity, functional MRI, prefrontal cortex, repetitive TMS

## Abstract

**Background:**

Recent evidence suggests that repetitive transcranial magnetic stimulation (rTMS) might be effective in treating generalized anxiety disorder (GAD). Cognitive models of GAD highlight the role of intolerance of uncertainty (IU) in precipitating and maintaining worry, and it has been hypothesized that patients with GAD exhibit decision‐making deficits under uncertain conditions. Improving understanding of the neural mechanisms underlying cognitive deficits associated with IU may lead to the identification of novel rTMS treatment targets and optimization of treatment parameters. The current report describes two interrelated studies designed to identify and verify a potential neural target for rTMS treatment of GAD.

**Methods:**

Study I explored the integrity of prefrontal cortex (PFC) and amygdala neural networks, which underlie decision making under conditions of uncertainty, in GAD. Individuals diagnosed with GAD (*n *=* *31) and healthy controls (*n *=* *20) completed a functional magnetic resonance imaging (fMRI) gambling task that manipulated uncertainty using high versus low error rates. In a subsequent randomized‐controlled trial (Study II), a subset of the GAD sample (*n *=* *16) completed the fMRI gambling task again after 30 sessions of active versus sham rTMS (1 Hz, right dorsolateral prefrontal cortex) to investigate the modulation of functional networks and symptoms.

**Results:**

In Study I, participants with GAD demonstrated impairments in PFC‐PFC and PFC‐amygdala functional connectivity (FC) mostly during the high uncertainty condition. In Study II, one region of interest pair, dorsal anterior cingulate (ACC) – subgenual ACC, showed “normalization” of FC following active, but not sham, rTMS, and neural changes were associated with improvement in worry symptoms.

**Conclusions:**

These results outline a possible treatment mechanism of rTMS in GAD, and pave the way for future studies of treatment optimization.

## 1 INTRODUCTION

Preliminary research has demonstrated that repetitive transcranial magnetic stimulation (rTMS) improves generalized anxiety disorder (GAD) symptoms (Bystritsky, Kerwin, & Feusner, 2009; Bystritsky et al., 2008; Diefenbach et al., 2016). rTMS uses cortical stimulation to modify neural activity locally, and perhaps more significantly, in large, diffused neural networks (Rossini et al., 2015; Wagner, Rushmore, Eden, & Valero‐Cabre, 2009). Thus, identifying the neural architecture and neuromodulation mechanism of action is crucial to optimizing rTMS treatments (Neggers, Petrov, Mandija, Sommer, & van den Berg, 2015). However, these have not yet been explored in GAD.

Patients with GAD are characterized by abnormal neural *activity*, in prefrontal cortical (PFC) and limbic regions, including anterior cingulate cortex (ACC), anterior insula (AI) and amygdala (Hilbert, Lueken, & Beesdo‐Baum, 2014; Mochcovitch, da Rocha Freire, Garcia, & Nardi, 2014; Taylor & Whalen, 2015). PFC‐amygdala *coupling* (or *functional connectivity* (FC); Friston, 2011), has also been shown to be weaker in GAD, and has demonstrated association with symptom severity. These findings suggest a decrease in top‐down inhibition of PFC on amygdala (Hilbert et al., 2014; Mochcovitch et al., 2014; Taylor & Whalen, 2015).

It is important to consider these neuronal abnormalities in the context of cognitive and emotional processes. A leading theory of GAD suggests a central role of “intolerance of uncertainty” (IU), a cognitive bias which interferes with information processing, including decision‐making (DM) (Ladouceur, Talbot, & Dugas, 1997). The IU model proposes that excessive emotional response in uncertain situations contributes to the development and maintenance of worry (Dugas, Gagnon, Ladouceur, & Freeston, 1998). Meta‐analytic research supports the IU model (Gentes & Ruscio, 2011), and IU has been found to predict GAD severity (Dugas et al., 2007). Effective DM requires salience processing, error monitoring, and emotion regulation (Clore & Huntsinger, 2007; Grecucci, Giorgetta, Van’t Wout, Bonini, & Sanfey, 2013), thus, deficits in any of these processes may contribute to the development and maintenance of GAD.

Many of the neural areas believed to underlie these DM processes have also been found to be impaired in patients with GAD. Dorsolateral prefrontal cortex (DLPFC), dorsal, rostral, and subcallosal (including subgenual) ACC (dACC, rACC, sgACC), and orbitofrontal cortex (OFC) are engaged during DM under uncertainty (Krain, Wilson, Arbuckle, Castellanos, & Milham, 2006); dACC, rACC, sgACC, and AI are activated during salience processing and error monitoring (Barch et al., 2001; Botvinick, Cohen, & Carter, 2004; Braver, Barch, Gray, Molfese, & Snyder, 2001; Menon & Uddin, 2010; Uddin, 2015; Ullsperger & von Cramon, 2001); and ACC, AI and amygdala are involved in emotion regulation (Ochsner, Silvers, & Buhle, 2012). Importantly, coupling of these regions comprises proposed neural networks underlying the cognitive‐emotional processes involved in DM (Khani & Rainer, 2016; Rushworth, Kolling, Sallet, & Mars, 2012).

Integrity of these networks in GAD has mostly been explored in the context of emotion dysregulation; however, the neural mechanism of IU per se has not been established. Previous research has found that, unlike healthy control (HC) adults, those diagnosed with GAD experience decreased amygdala activation during a high versus low certainty gambling task (Yassa, Hazlett, Stark, & Hoehn‐Saric, 2012). In addition, AI activations during an ambiguous affective DM task are significantly associated with self‐reported IU in an unselected sample of young adults (Simmons, Matthews, Paulus, & Stein, 2008). Importantly, no studies have reported FC analysis of DM under uncertainty in GAD.

This report describes two interrelated studies. Study I aimed to characterize the *neural circuit FC* underlying the cognitive processes related to DM under uncertainty, focusing on fronto‐limbic FC, in patients with GAD versus HCs. We predicted that individuals with GAD would demonstrate weaker PFC‐amygdala FC, evidencing less inhibition of emotional responses, and stronger reactivity of cognitive‐emotional error monitoring and salience PFC circuit (i.e., ACC and AI) during high uncertainty trials (i.e., trial blocks involving high rates of error feedback or “lose” trials). Further we predicted that FC during high uncertainty trials would correlate with trait measures of GAD symptoms (i.e., worry) and IU.

Study II aimed to demonstrate modulation of fronto‐limbic circuit FC following rTMS treatment. In a randomized control trial (RCT) we previously showed that, in GAD, right DLPFC‐targeted low‐frequency rTMS, but not sham, improved anxiety, worry and depressive symptoms and altered local DLPFC *activation* during a gambling DM task under conditions of uncertainty (Diefenbach et al., 2016). Since DLPFC, which has been implicated in GAD (e.g., Hilbert et al., 2014) is part of the DM network (Krain et al., 2006) and rTMS is believed to alter neural networks architecture (i.e., FC; Rossini et al., 2015; Wagner et al., 2009), we test the hypothesis that *FC patterns* during high uncertainty trials would normalize following treatment with active versus sham rTMS to this region, and that changes in FC would correlate with improvements in symptoms and IU trait in GAD participants receiving active rTMS.

## 2 MATERIALS & METHODS

### 2.1 Study I

#### 2.1.1 Participants

Fifty‐one adults (≥18 years old) completed the fMRI gambling task during participation in either a single session neuroimaging study or during the baseline assessment of a randomized‐controlled trial (Clinical Trials ID: NCT01607710). Participants in the GAD group (*n *=* *31) were diagnosed with either principal or coprincipal GAD of at least moderate severity (Clinical Global Impression‐Severity (Guy, 1976) ≥4) with Hamilton Anxiety Rating Scale (HARS; Shear et al., 2001) ≥18 and 17‐item Hamilton Rating Scale for Depression (HRSD; Williams, 1988) ≤17. Psychiatric exclusions for the GAD group included post‐traumatic stress disorder (current), substance use disorder (past 6 months); or lifetime bipolar, psychotic, developmental, or obsessive‐compulsive disorder. Participants taking psychiatric medications were enrolled so long as pharmacotherapy was stabilized for 3 months prior to study entry, with the exception of benzodiazepines taken as needed, which were stabilized based upon medication half‐life. Participants enrolled in the HC group (*n *=* *20) reported no current psychiatric diagnoses or lifetime psychiatric treatment. Participants in both groups were excluded for medical disorders which could confound imaging (e.g., brain trauma) or situations that were unsafe (e.g., metal in body). While there was no a priori IQ exclusion, all participants were assessed to have an estimated IQ >80 (measured by NeuroTrax^™^ Comprehensive Testing Suite global cognitive score; NeuroTrax Corp., Bellaire, TX).

#### 2.1.2 Measures

Inclusion criteria were confirmed with the Mini International Neuropsychiatric Interview (Sheehan et al., 1998), Clinical Global Impression‐Severity scale (Guy, 1976), structured interview guides for the HARS (Shear et al., 2001) and 17‐item HRSD (Williams, 1988), administered by either a licensed psychologist or a Masters‐level research assistant under supervision of a licensed psychologist. Trait worry and IU were assessed using The Penn State Worry Questionnaire (PSWQ; Meyer, Miller, Metzger, & Borkovec, 1990) and Intolerance of Uncertainty Scale (IUS; Freeston, Rhéaume, Letarte, Dugas, & Ladouceur, 1994) respectively. Both the PSWQ and IUS are well‐validated and sensitive to treatment effects (Antony, Orsillo, & Roemer, 2001; Bomyea et al., 2015; Stanley et al., 2003). In both measures higher scores indicate more severe psychopathology.

#### 2.1.3 Functional MRI task

During a computerized gambling task, adapted from Bystritsky et al. (2008) and described in our previous report (Diefenbach et al., 2016), participants were shown two cards (red and blue) and asked to predict which card would be drawn next. Participants were instructed to “look for a pattern.” Unknown to them, trials were presented in Win and Lose Blocks, in which 75% of the trials showed participants correct or error feedback respectively. Thus, lose blocks constitutes a ‘high uncertainty’ condition, given that significantly more error feedback is presented. Each condition (Win/Lose) included six blocks with eight trials/block, with win/lose trials presented randomly. Trials were presented for 2.3 s each with feedback (correct or error) presented for 1.2 s (task block length = 28 s). Rest blocks showing a white cross over a black background for 18 s in length interleaved task blocks (total run length = 381 s, including 13 s of instructions). Before the task, participants were given 50 points (with no monetary value) and told that they could win or lose two points per trial based upon correct or incorrect predictions respectively. By design, all participants ended with a loss of 16 points.

#### 2.1.4 Image acquisition

MRI scans were conducted on a Siemens 3T Allegra MRI scanner. Blood oxygenation level dependent (BOLD) contrast was obtained with the following T2*‐weighted echo planar imaging (EPI) sequence: TR/TE = 1,860/27 ms, Flip angle = 70°, FOV = 22 cm, 64 × 64 acquisition matrix with thirty‐six contiguous axial slices 3 mm thick (1 mm gap), yielding 3.4 × 3.4 × 4 mm voxels. Overall, 208 images were acquired, starting with 7 “dummy” images, which were excluded from analysis.

#### 2.1.5 Data analysis

##### Regional activation analysis

Imaging data were analyzed using SPM8 (Wellcome Department of Cognitive Neurology, London, UK). Each individual’s dataset was realigned to the first “nondummy” image using the INRIAlign toolbox (A. Roche, INRIA Sophia Antipolis, EPIDAURE Group) to correct for head motion, spatially normalized to the Montreal Neurological Institute (MNI) space (Karl J. Friston et al., 1995) and smoothed with a 5 mm isotropic (FWHM) Gaussian kernel. A high‐pass filter with a cut‐off of 128 s was applied to correct for signal low‐frequency drift.

A general linear model (GLM) was calculated for each participant with the Task Condition (Win/Lose) blocks regressors modeled as boxcar functions convolved with the SPM8 canonical hemodynamic response function (HRF). Individual statistical parametric maps were calculated for each of the conditions to be used in group analyses, as described next.

To assess brain regions functionally involved with the task (i.e., defining regions of interest; ROIs), individual statistical maps were entered into a mixed‐effect repeated measures analysis of variance (ANOVA) with Task Condition (Win/Lose) as the within‐subject effect and Group (GAD/HC) as the between‐subject effect. While Task Condition effects were the primary focus for ROI definition (see below), Group main effect, as well as Group by Task Condition interaction were also explored.

##### ROI‐to‐ROI functional connectivity analysis

Functional connectivity analysis was performed using Functional Connectivity (CONN) toolbox version 14.n (http://web.mit.edu/swg/software). Preprocessing was redone using CONN’s standard pipeline, including: realignment, coregistration with a high‐resolution anatomic scan, slice time correction, structural segmentation, normalization to MNI template, and smoothing (FWHM 8 mm^3^). White matter and cerebrospinal fluid were computed per subject, and entered as potential confound regressors along with realignment effects and scrubbing parameters (set according to CONN defaults: global‐signal scan‐to‐scan *Z*‐value = 9; motion threshold = 2 mm). Task Conditions (win/lose for Study I and II) and Time (pre/post rTMS for Study II) were entered as within‐subjects regressors of interest while Group (GAD vs. HC for Study I) and Treatment Condition (active vs. sham rTMS for Study II) were entered as between‐subjects regressors of interest, using CompCor (Behzadi, Restom, Liau, & Liu, 2007). Band‐pass filter (0.008–0.09 Hz) was applied, followed by detrending (removal of linear trends within each functional session), to reduce noise influence.

##### ROIs definition

As mentioned above, brain regions functionally involved in the task were defined as having a significant Task Condition effect in the group activation analysis of Group by Task Condition ANOVA. Spheres, 5 mm in diameter around point of maximal group activation, were defined and entered into CONN as ROIs. For ROIs not identified by GLM analysis binary masks were created based on the FSL Harvard‐Oxford atlas (Desikan et al., 2006).

##### Functional connectivity analysis

Individual (first‐level) ROI‐to‐ROI FC analysis was performed by calculating the time courses temporal weighted‐correlations for all pair‐wise ROI combinations. Next, these measures were entered into repeated‐measures ANOVA across subjects (second‐level analyses) using a standard mixed within‐ (Task Condition) and between‐ subjects (Group) GLM, as described above. Significant results were considered at FDR corrected *p* value (*q*_FDR_) < 0.05.

To assess the relationship between GAD psychopathology and ROI‐to‐ROI FC patterns, correlation analyses were performed for each of the Task Conditions separately. Correlation of FC with PSWQ and IUS were first calculated in the entire sample and interpreted as significant at *p *<* *0.0125, applying correction for each ROI pair for four comparisons (two measures for each of the two conditions). Follow‐up exploratory correlation analyses within the GAD group were conducted for significant results at the entire‐sample level.

### 2.2 Study II

#### 2.2.1 Participants/Image acquisition

In Study II we present data from a GAD subgroup who completed the fMRI task a second time after a treatment course of active (*n *=* *9) or sham (*n *=* *7) rTMS (*M *=* *6.06 ± 3.3, range = 1–12 days between final rTMS session and second fMRI). In addition to the exclusion criteria outlined in Study I, participants were also excluded from Study II for concurrent psychotherapy. Therefore, no participants in Study II were undergoing psychotherapy over the course of rTMS treatment. In addition, for those participants taking psychiatric medication, type and dose remained stable over the course of rTMS treatment.

Image acquisition parameters were identical to Study I.

#### 2.2.2 rTMS protocol

Participants completed 30 sessions (5 days/week for 6 weeks) of low‐frequency (1 Hz; 90% of the resting motor threshold) rTMS for 900 pulses/session. These stimulation parameters were chosen to be the same as those used in a previous open trial of rTMS for GAD (Bystritsky et al., 2008), although a longer treatment course (i.e., 30 sessions) was administered in the current study to protect against inadequate dosing. rTMS was administered using the FDA‐Cleared Neurostar TMS Therapy System (note that neither the use in GAD nor the protocol used here are FDA approved), and sham rTMS was administered using a sham coil (Neuronetics XPLOR) that delivers <10% of an active pulse. rTMS was administered to the right DLPFC (MNI coordinates: *x *=* *42, *y *=* *36, *z *=* *32) using stereotactic neuronavigation system (Visor2, ANT Neuro, Enschede, Netherlands; http://www.ant-neuro.com), as described previously (Diefenbach et al., 2016).

#### 2.2.3 Data analysis

Since no group effects were found between GAD and HC with GLM *activation* analysis in Study I (see Results), only FC analysis was performed for Study II, to assess the effects of active (vs. sham) rTMS on the ROIs pairs showing abnormal *FC* in GAD compared to HC in Study I.

Individual ROI‐to‐ROI FC analysis was calculated as described above for pre‐ and post‐treatment scans. Next, Treatment Condition (Active/Sham) by Time (Pre/Post‐treatment) repeated measures ANOVAs were calculated for either the Lose or Win task conditions separately, based on a‐priori hypotheses and results from Study I, as described below. Due to relatively small sample size, threshold was set at uncorrected *p *<* *0.05, and effect sizes are also presented to aid interpretation.

To assess the relationship between pre‐to‐post‐treatment changes in brain functional architecture and GAD psychopathology, correlations between FC changes and IUS and PSWQ changes over time (post‐pre) were calculated for the active rTMS group.

## 3 RESULTS

### 3.1 Study I

#### 3.1.1 Participants

Table 1 outlines demographic and symptom characteristics. Groups were matched on age, gender, race, and estimated IQ. GAD was the principal or coprincipal diagnosis in all patients; however, 19 (61%) met criteria for other anxiety or depressive disorders at the time of study participation. Twenty (64%) of GAD participants were undergoing pharmacological treatments (for detailed list of psychiatric comorbidity and pharmacological treatments see Supporting Information Table S1).

**Table 1:** Study I: Sample demographic and clinical characteristics

	GAD (*n *=* *31)	HC (*n *=* *20)	Statistics
Age (in years)	42.35 ± 14.3	39.75 ± 15.5	*t *=* *0.6, *p *=* *0.5
Gender (M/F)	7/24	6/14	χ^2^ = 0.3, *p *=* *0.5
Estimated IQ	100.56 ± 6.9	102.43 ± 8.4	*t *=* *0.8, *p *=* *0.4
Race (W/AA/A/NK)	29/0/1/1	18/0/2/0	χ^2^ = 0.9, *p *=* *0.3
HARS	22.3 ± 4.8	0.3 ± 0.6	*t *=* *20.4, *p *<* *.0001
HRSD	13.5 ± 3.2	0.3 ± 0.7	*t *=* *18.2, *p *<* *.0001
PSWQ^a^	67.6 ± 8.3	34.3 ± 9.5	*t *=* *13.1, *p *<* *.0001
IUS^a^	86.1 ± 19.5	44.9 ± 14.3	*t *=* *8.1, *p *<* *.0001
Psychiatric co‐morbidity^b^			
Any psychiatric diagnosis	19/31	—	
Depression	12/31	—	
Other anxiety disorder	11/31	—	
Psychiatric pharmacotherapy^b^			
Any psychiatric medication	20/31	—	
Anti‐depressant	13/31	—	
Anxiolytics (including benzodiazepines)	13/31	—	

*Note*

Race: W: White, AA: African American; A: Asian; NK: Not known; HARS: Hamilton Anxiety Rating Scale; HRSD: Hamilton Rating Scale for Depression; PSWQ: Penn State Worry Questionnaire; IUS: Intolerance of Uncertainty Scale; ^a^Scores available for 30/31 GAD and 20/20 HC; ^b^Detailed information on psychiatric diagnosis and treatment per participant is provided in Supporting Information Table S1.

#### 3.1.2 GLM activation results

Repeated measures ANOVA with a between‐subject factor of Group (GAD/HC) and within‐subject factor of Task Condition (Win/Lose) revealed a significant main effect of Task Condition in the following PFC regions: Bilateral DLPFC, dACC, AI and pre‐SMA (*q*_[FDR]_ < 0.05; Supporting Information Figure S1 provides enlarged maps for all regions and Table 2 details coordinates and statistical results). Post‐hoc analyses demonstrated that the main effect of Task Condition was due to stronger activations during Lose versus Win blocks across all regions. In contrast, only sgACC showed stronger activation in Win versus Lose at *p*_(uncorrected)_ < 0.01 (Table 2). It is important to highlight that no region showed an *activation* main effect of Group or Group by Task Condition interaction for analyses of activation. These task‐related ROIs were therefore used for subsequent *functional connectivity* analyses exploring a‐priori hypothesized group effects, as the absence of a Group or Group by Task Interaction in the initial activation analysis minimizes introducing an ROI selection bias in the FC analyses.

**Table 2:** Study I: Prefrontal cortex (PFC) activations during the gambling decision‐making task

Anatomic location of maximum activation	MNI coordinates			*F* score
	*x*	*y*	*z*	
Main effect of Condition (*q*_FDR_ < 0.05; Lose > Win)				
dACC1	0	26	40	15.7
dACC2	−9	20	34	22.49
Right AI	33	20	4	16.98
Left AI	−36	14	7	16.33
Right DLPFC	39	50	31	27.51
Left DLPFC	−36	50	28	20.18
Pre‐SMA	3	8	55	15.93
T‐test: Win > Lose (*p *<* *0.01)				T score
sgACC	6	35	−17	2.77

*Note*

AI: anterior insula; dACC: dorsal anterior cingulate cortex; DLPFC: dorsolateral prefrontal cortex; sgACC: sub‐genual ACC; SMA: supplementary motor cortex.

#### 3.1.3 Functional connectivity analysis

We focused our FC analysis on PFC regions and amygdala given their documented role in IU (Krain et al., 2006). PFC ROIs were defined as spheres at regions showing a Task Condition main effect in GLM analysis (Table 2). Since no activation effects were found in the amygdala, right and left amygdala masks were created based on the Harvard‐Oxford atlas (right amygdala coordinates: *x *=* *28, *y * =  −2, *z * =  −24; left amygdala: *x * =  −22, *y * =  −6, *z * =  −20; see Supporting Information Figure S1 for ROIs maps).

**Figure 1:**
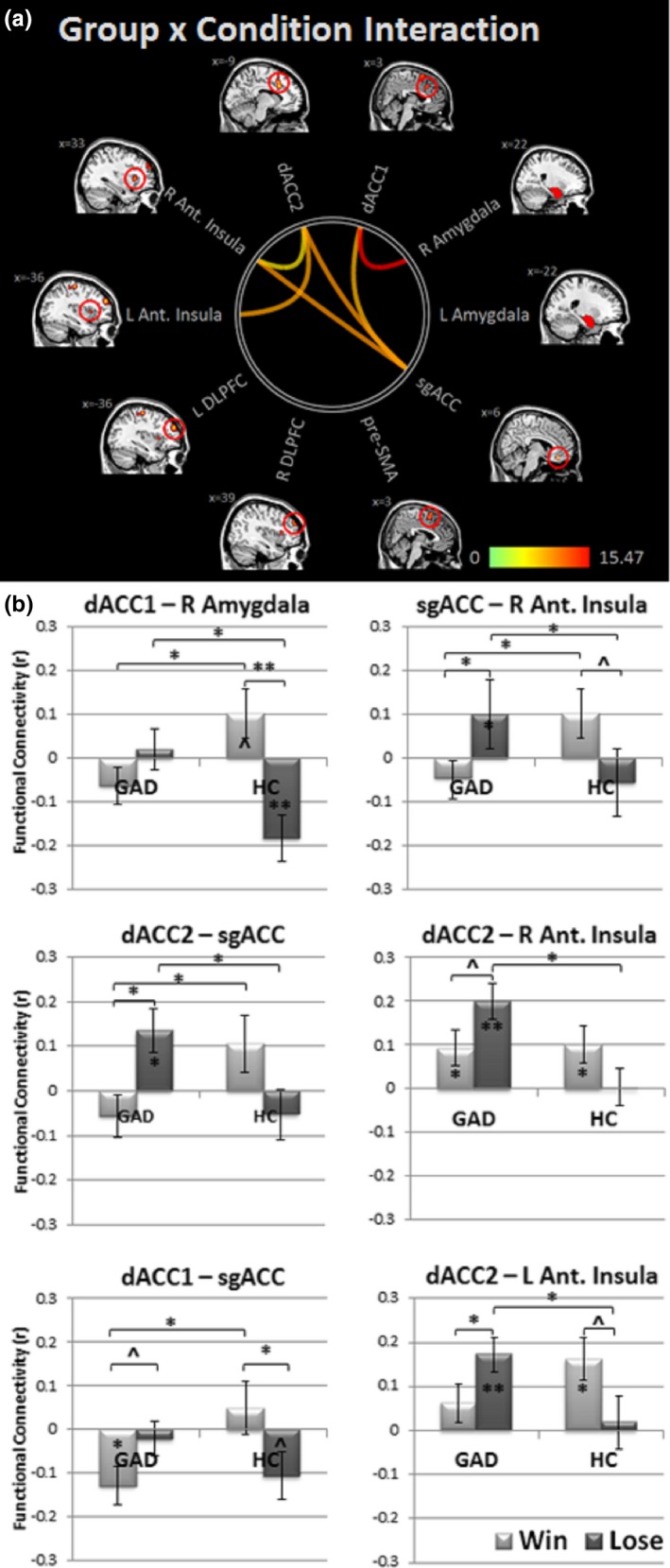
Study I: Functional connectivity results. Panel (a) depicts ROI pairs showing significant Group (GAD vs. HC) by Task Condition (Win vs. Lose blocks) interaction (*q*_FDR_ < 0.05). Panel (b) presents post‐hoc effects for significant results. **p <* *0.001; *p <* *0.05; ^^^ *p *<* *0.08

A Group (GAD/HC) by Task Condition (Win vs. Lose) repeated measures ANOVA (*q*_FDR_ < 0.05) revealed a significant Group by Task Condition interaction in FC between (a) dACC1 and right Amygdala, (b) dACC2 and sgACC, (c) dACC1 and sgACC, (d) sgACC and right AI, (e) dACC2 and right AI, and (f) dACC2 and left AI; see Figure 1a and Table 3 for statistical results. No ROI‐pair FC showed a significant main effect of Group or Task Condition at *q*_FDR_ < 0.05.

**Table 3:** Study I: Statistical results for group × task condition ANOVA

	Group × task condition ANOVA		Post‐hoc *t*‐tests								
	G × TC interaction			GAD			HC			GAD vs. HC	
	*F*_(1,49)_	*q*_FDR_		FC	*t*_(1,30)_	*p*	FC	*t*_(1,19)_	*p*	*t*_(1,49)_	*p*
dACC1‐right Amygdala	15.47	0.002	Win	−0.06 ± 0.2	−1.49	>0.1	0.1 ± 0.2	1.85	0.08	2.38	0.02
			Lose	0.02 ± 0.2	0.45	>0.1	−0.18 ± 0.2	−3.37	0.003	−2.81	0.007
dACC2‐sgACC	9.60	0.02	Win	−0.05 ± 0.2	−1.19	>0.1	0.10 ± 0.3	1.63	>0.1	2.78	0.009
			Lose	0.14 ± 0.3	2.71	0.01	−0.05 ± 0.2	−0.92	>0.1	−2.43	0.02
dACC1‐sgACC	8.21	0.03	Win	−0.13 ± 0.2	−2.86	0.008	0.05 ± 0.3	0.81	>0.1	0.30	>0.1
			Lose	−0.02 ± 0.2	−0.55	>0.1	−0.11 ± 0.2	−1.96	0.06	1.23	>0.1
sgACC‐right AI	9.58	0.03	Win	−0.45 ± 0.2	−1.14	>0.1	0.10 ± 0.3	1.76	0.093	2.17	0.03
			Lose	0.10 ± 0.3	2.11	0.04	−0.06 ± 0.2	−1.01	>0.1	−2.11	0.04
dACC2‐right AI	6.25	0.05	Win	0.09 ± 0.2	2.29	0.03	0.10 ± 0.2	2.46	0.02	0.13	>0.1
			Lose	0.20 ± 0.2	5.04	0.00002	0.003 ± 0.2	0.07	>0.1	−3.26	0.002
dACC2‐left AI	8.84	0.03	Win	0.06 ± 0.2	1.44	>0.1	0.16 ± 0.2	3.35	0.003	1.05	>0.1
			Lose	0.17 ± 0.2	4.33	0.0001	0.02 ± 0.3	0.30	>0.1	2.52	>0.1

*Note*

FC: functional connectivity value (mean ± *SD*); AI: anterior insula; dACC: dorsal anterior cingulate cortex; G: group; GAD: generalized anxiety disorder; HC: healthy controls; sgACC: SUB‐genual ACC; TC: task condition.

Figure 1b and Table 3 display post‐hoc analyses (Supporting Information Figure S2 provides full FC maps for each group and task condition). Results indicated significant positive FC for dACC2‐sgACC, dACC2‐right AI, dACC2‐left AI, and sgACC‐right AI during Lose in the GAD group only, with significant group differences. In addition, dACC1‐right Amygdala FC was significantly anti‐correlated in HCs, but not GAD during Lose blocks, with significant group difference. dACC1‐right Amygdala FC also differed significantly between HC and GAD groups during Win; however, FC did not differ significantly from zero (i.e., no significant correlation between these two regions) for either group. dACC2‐sgACC and sgACC‐right AI also showed significant group differences during Win; again, FC did not differ significantly from zero. Finally, for dACC1‐sgACC, only the GAD group showed significant anticorrelation during Win with significant group difference, while the HC group showed a trend toward anticorrelation during Lose (group difference during Lose was not statistically significant).

**Figure 2:**
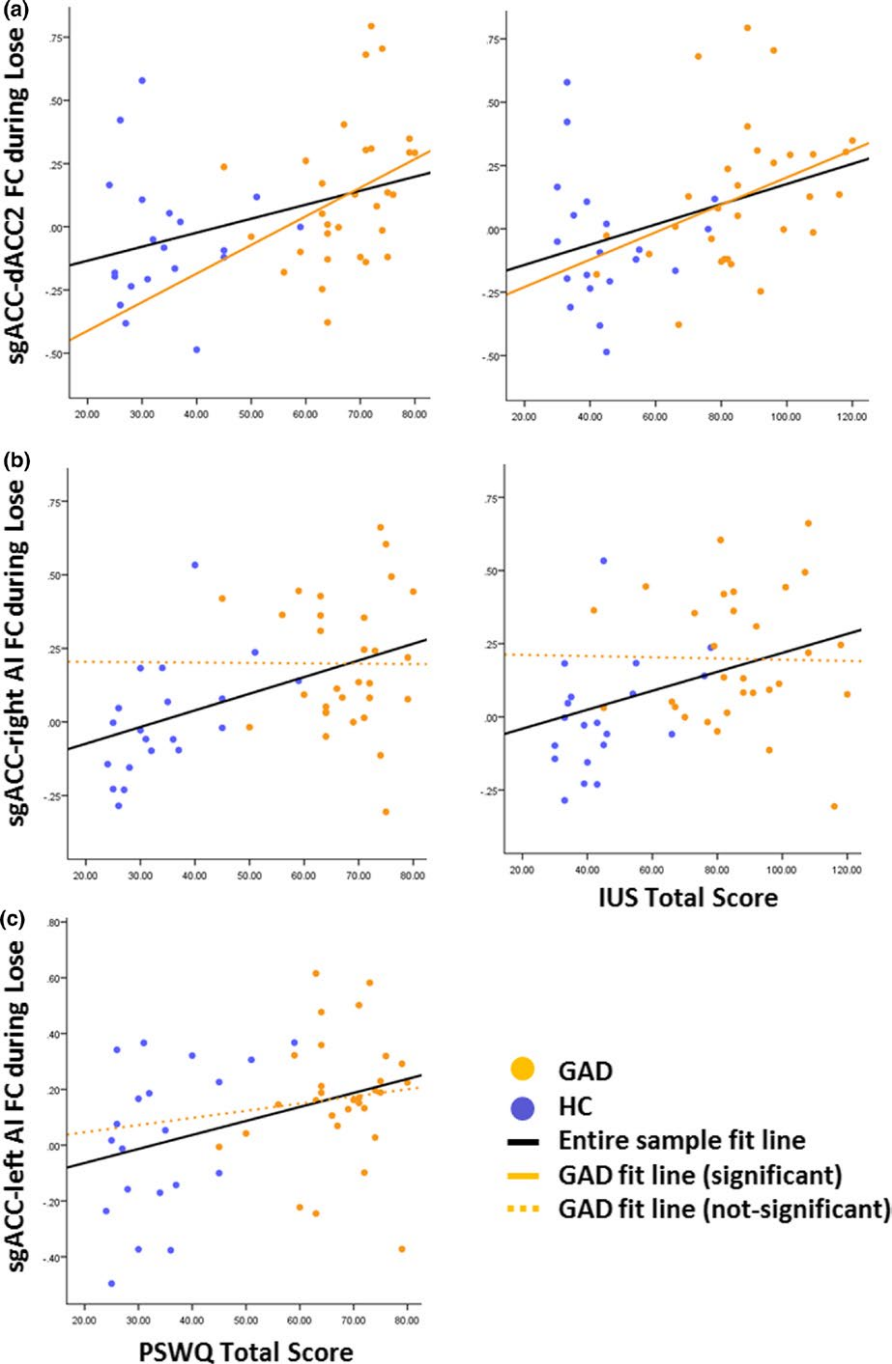
Study I: Correlations between functional connectivity and psychopathology symptoms. Results are shown only for ROI pairs and Conditions with significant correlation on the entire sample level (black fit line): (a) sgACC‐dACC2, (b) sgACC‐right anterior insula (AI), and (c) sgACC‐left AI. Post‐hoc correlations in the GAD group are depicted with orange fitted line (non‐significant correlations in dashed line). IUS: Intolerance of Uncertainty Scale; PSWQ: The Penn State Worry Questionnaire

Correlation analyses in the combined sample indicated significant associations between FC (specifically dACC2 FC) and GAD psychopathology symptoms during Lose blocks only (Figure 2). Results indicated that dACC2‐sgACC FC correlated positively and significantly with IU (IUS; *r *=* *0.38, *p *=* *0.006), and trait worry (PSWQ; *r *=* *0.37, *p *=* *0.009). dACC2‐right AI FC also correlated positively and significantly with IUS (*r *=* *0.37, *p *=* *0.007) and PSWQ (*r *=* *0.46, *p *=* *0.001), while dACC2‐left AI FC correlated positively and significantly with PSWQ only (*r *=* *0.37, *p *=* *0.008). The other three ROI pairs (dACC1‐right amygdala, dACC1‐sgACC and sgACC‐right AI) did not show significant correlations with either IUS or PSWQ in the combined sample. Exploratory correlation analyses within the GAD sample indicated a positive association between dACC2‐sgACC FC and GAD psychopathology, which reached statistical significance for IUS (*r *=* *0.37, *p *=* *0.04), but not for PSWQ (*r *=* *0.34, *p *=* *0.07). No other ROI FC pairs were associated with GAD psychopathology in this group.

### 3.2 Study II

#### 3.2.1 Participants

Characteristics of the participants in Study II are described in Table 4. The groups were matched on age, gender, race, estimated IQ and symptom severity. As we reported previously (Diefenbach et al., 2016), participants in the active rTMS group showed significantly more pre‐to‐post‐treatment effect on symptoms with 7 (77.8%) compared with 2 (28.6%) meeting treatment responder status (defined as ≥50% HARS improvement) (χ^2^_[1, *N* = 16]_ = 3.87, *p *=* *0.049).

**Table 4:** Study II: Sample demographic and symptom characteristics

	Active rTMS (*n *=* *9)	Sham rTMS (*n *=* *7)	Statistics
Age (in years)	46.44 ± 10.8	39.71 ± 16.0	*t *=* *−1.0, *p *=* *0.3
Gender (M/F)	1/8	2/5	χ^2^ = 0.8, *p *=* *0.4
Estimated IQ	102.46 ± 8.4	99.34 ± 6.6	*t *=* *−0.8, *p *=* *0.4
HARS	24.9 ± 5.2	21.3 ± 4.3	*t *=* *1.5, *p *=* *0.2
HRSD	14.7 ± 3.4	13.6 ± 1.9	*t *=* *0.8, *p *=* *0.5
PSWQ	69.7 ± 5.4	64.5 ± 11.5	*t *=* *1.2, *p *=* *0.3
IUS	82.4 ± 15.5	80.9 ± 20.6	*t *=* *0.2, *p *=* *0.9

*Note*

HARS: Hamilton Anxiety Rating Scale; HRSD: Hamilton Rating Scale for Depression; PSWQ: Penn State Worry Questionnaire; IUS: Intolerance of Uncertainty Scale.

#### 3.2.2 Functional connectivity analysis

Analyses for Study II examined treatment effects on ROI pairs that were significantly related to GAD status (i.e., showed significant FC Group effects) in Study I (Table 3). To test our a priori hypotheses we focused Study II analyses on the Lose condition. This choice is further supported by results from Study I indicating that most statistically significant FC results were found during this condition. One exception was the dACC1‐sgACC FC pair where a group difference was found for the Win condition. Thus, we explored treatment effects during the Win condition for the dACC1‐sgACC pair only.

**Figure 3:**
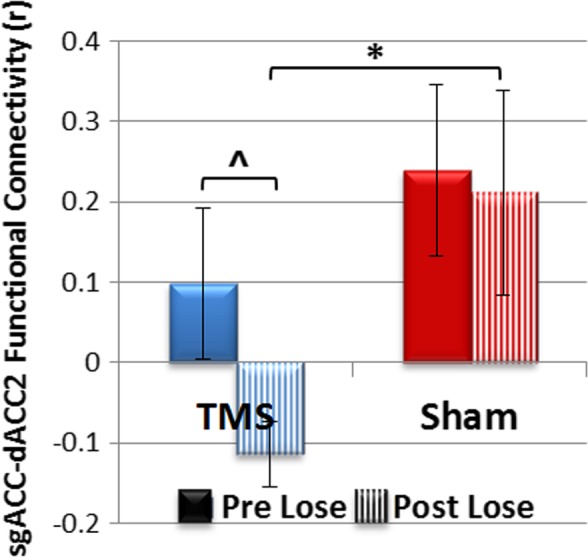
Study II: Bar graph depicting pre‐to‐post active versus sham rTMS effect during the lose condition for sgACC‐dACC functional connectivity. *p =* *0.01, ^*p *=* *0.07

A Treatment Condition (Active/Sham) by Time (Pre vs. Post) ANOVA in the Lose condition revealed a main effect of treatment condition in dACC2‐sgACC FC (*F*_[1,14]_ = 5.17, *p *=* *0.039; Figure 3). There was no significant main effect of Time or Treatment Condition by Time interaction. Exploratory post‐hoc analysis demonstrated that patients receiving active rTMS differed significantly from those receiving sham in dACC2‐sgACC FC only at post‐treatment (*t *=* *2.7, *p *=* *0.01, *d*′ = 1.28). Specifically, patients receiving active rTMS showed negative dACC2‐sgACC FC at post‐treatment (a moderate decrease from baseline; paired *t* test: *t*_(8)_ = 2.03, *p *=* *0.07, d′ = 0.68), while this FC was positive in patients receiving sham (with no Time effect; *p *>* *0.1; d′ = 0.07). Post rTMS FC in GAD was not significantly different than that of controls at baseline (*t*_[27]_ = −0.68, *p *=* *0.5). No other ROI‐pairs showed significant Group or Time main effects or interaction in the conditions tested (a‐priori tested for the Lose condition for all six pairs outlined in Figure 1 and Table 3 and exploratory tested for Win condition for dACC1‐sgACC only. See Supporting Information Figure S3 and Table S2 for bar graphs and statistical results). Pre‐ to post‐treatment changes in dACC2‐sgACC FC were moderately, though nonsignificantly, associated with changes in trait worry (PSWQ *r *=* *0.53) and minimally associated with changes in IU (IUS *r *=* *0.22) in the active rTMS group.

## 4 DISCUSSION

We aimed to outline the abnormal neural network architecture in GAD during uncertain DM and the potential role of neuromodulation for altering these circuits. Results indicated that GAD is associated with a decrease in negative (anti‐) correlation between dACC (cluster #1) and amygdala, and an increase in positive correlations between insular and prefrontal regions (dACC2‐sgACC, dACC2‐bilateral AI, sgACC‐right AI). Further, right DLPFC‐targeted rTMS modified the dACC‐sgACC FC in the direction of “normalization.”

The frontal regions that showed differential FC between GAD and HCs during high error feedback trials (i.e., dACC, sgACC, AI, DLPFC), are involved in salience identification, error monitoring and emotional control (Botvinick et al., 2004; Menon & Uddin, 2010; Ochsner et al., 2012; Uddin, 2015). Decreased PFC‐amygdala connectivity is perhaps the most consistent *FC* abnormality reported for these regions in GAD (Hilbert et al., 2014; Mochcovitch et al., 2014; Taylor & Whalen, 2015). In HCs these regions are anticorrelated, which is often interpreted as PFC inhibiting amygdala (Banks, Eddy, Angstadt, Nathan, & Phan, 2007; Etkin, Egner, Peraza, Kandel, & Hirsch, 2006; Kim et al., 2011; Ochsner et al., 2012). Our results similarly indicate an absence of dACC‐amygdala anti‐correlation in GAD versus HC during DM. In addition, results indicated increased frontal FC in GAD for core areas of the salience network (SN, i.e., dACC‐AI) (Menon & Uddin, 2010; Uddin, 2015), potentially indicating a hypervigilant state in GAD. Sub‐genual ACC is also involved in emotion modulation (Delgado, Nearing, Ledoux, & Phelps, 2008; Diekhof, Geier, Falkai, & Gruber, 2011; Drevets, Savitz, & Trimble, 2008; Glascher et al., 2012; Urry et al., 2006) and evidenced more positive FC with both cognitive control (i.e., dACC) and emotional (i.e., right AI) circuits in GAD. This, in combination with decreased PFC‐amygdala inhibition, potentially leads to increased emotional reactivity. Interestingly, increased frontal, but not PFC‐amygdala, FC correlated with traits of worry and IU, suggesting a possible endophenotype for GAD‐related psychopathology.

Study II’s results indicated that right DLPFC‐targeted rTMS modifies dACC‐sgACC FC in patients with GAD in the direction of “normalization” (from positive to negative FC). Further, post‐rTMS changes in dACC‐sgACC FC were moderately associated with improvements in worry symptoms, although this result should be carefully interepreted as it was not significant (this can be attributed to low power but previous work also had not shown correlation between FC and symptom changes following rTMS; Liston et al., 2014). These results are consistent with literature implicating the sgACC as a key structure in neuromodulation therapies for emotional disorders. The sgACC is a common deep brain stimulation target for major depressive disorder (MDD; Mayberg et al., 2005) as well as a proposed downstream mechanism through which cortical stimulation from rTMS improves symptoms (Pathak, Salami, Baillet, Li, & Butson, 2016). Previous research has also indicated that DLPFC‐targeted rTMS decreases sgACC resting‐state activity (Baeken et al., 2015; Fox, Halko, Eldaief, & Pascual‐Leone, 2012; Noda et al., 2015) and FC with several brain areas in patients with MDD (Baeken et al., 2014; Liston et al., 2014; Taylor et al., 2018) and post‐traumatic stress disorder (PTSD; Philip et al., 2018). Although preliminary, current findings suggest that sgACC, and specifically its FC with dACC, is a potential target for rTMS treatment for GAD as well.

As hypothesized, dACC‐sgACC FC changes were associated with improvements in worry; however, contrary to hypothesis, not with changes in IU. This is surprising given previous research indicating that changes in IU may mediate clinical symptom improvements in GAD following psychological therapies (Bomyea et al., 2015). It is possible that neurostimulation such as rTMS may exert GAD treatment effects through a different mechanism. This interpretation, and lack of rTMS influence on other ROI pairs FC, should be taken cautiously, as Study II’s small sample size undermines our ability to interpret negative results.

Notably, FC of only one ROI pair demonstrated group effect under conditions of high rates of correct feedback during decision‐making (i.e., Win). Specifically, significant dACC1‐sgACC anti‐correlation was found in GAD only. No a priori hypotheses were put forth for the Win condition. However, sgACC is also involved in processing and prediction of positive emotions (Manohar & Husain, 2016), including evaluation of rewards value (Levy & Glimcher, 2012). Thus, abnormally increased negative FC between these two emotion‐cognitive control regions might indicate downregulation of (or low reactivity to) positively‐valenced stimuli. This, in conjunction with hyper‐reactivity to negative stimuli, may contribute to information processing biases in GAD (Hayes & Hirsch, 2007).

### 4.1 Study limitations

We note several limitations of our studies. First, while GAD was the primary diagnosis in all patients, over half (61%) met criteria for other anxiety or depressive disorders, and there was significant correlation between depression and anxiety symptoms, limiting the specificity of the results. Second, the majority (64%) of GAD patients were undergoing pharmacological treatments, thus their secondary effect cannot be assessed. Third, Study II includes a small number of GAD patients undergoing either active or sham rTMS treatment, which dictated a liberal statistical threshold of uncorrected *p *<* *.05. Thus, our results should be considered preliminary and replicated with a larger sample. Finally, although the ROIs for FC analysis were identified using a unique analysis in terms of method (activation vs. FC) and contrast of interest (Task Condition main effect vs. Group by Task Condition interaction), the FC analyses nonetheless included an identical sample as that used to identify ROIs (study I), which may have affected independency (Kriegeskorte, Simmons, Bellgowan, & Baker, 2009).

### 4.2 Study summary

To summarize, we demonstrated functional neural networks architecture abnormalities, focusing on PFC and amygdala, during a DM under uncertainty task in GAD versus HC and their relationship to trait worry and IU. Results suggest increased emotional reactivity combined with decreased emotional and cognitive regulation during the task characterized by high error feedback is associated with a core symptom of GAD, i.e., excessive worry. Furthermore, a follow‐up RCT in a GAD subsample indicated that these abnormalities can be modulated by right DLPFC rTMS, leading to normalization of FC between key emotion regulation areas, sgACC and dACC, along with symptom improvement. These results outline a possible treatment mechanism, providing a target for future studies examining treatment optimization for GAD, preferably on an individualized level.

## CONFLICT OF INTEREST

Drs. Diefenbach and Goethe report that they receive material support from Neuronetics.

Dr. Goethe reports that he has received speaker fees to discuss TMS at professional conferences from Neuronetics and grant support from Astra Zeneca, Bristol‐Myers Squibb, Eli Lilly, Eisai, Forest, Hoffmann‐La Roche, Janssen, Johnson & Johnson, Merck/Schering‐Plough, Neuronetics, Neosync, Novartis, Otsuka, Pfizer, Shire Sunovion, Takeda, and Teva. Dr. Tolin receives funding from Palo Alto Health Sciences. Dr. Assaf, Dr. Rabany, Mr. Zertuche and Ms. Bragdon report no biomedical financial interests or potential conflicts of interest.
