# Supplementary material for: Neural functional architecture and modulation during decision making under uncertainty in individuals with generalized anxiety disorder
Source: Brain Behav. 2018 Jun 21;8(8):e01015. doi: 10.1002/brb3.1015 (PMC6085921; doi:10.1002/brb3.1015)
Supplement: Supplementary file 1 [file BRB3-8-e01015-s001.docx]

**Supplementary Materials**

**Supplementary Table 1.** Psychiatric co-morbidity and pharmacological treatments at time of study enrollment for individuals with GAD.

| Participant  # | Study II Treatment Group | Psychiatric Co-Morbidity | Pharmacological Treatment (Psychiatric drugs) |
| --- | --- | --- | --- |
| 1 | Study I only | None | Escitalopram, Bupropion, Lisdexamfetamine |
| 2 | Study I only | None | None |
| 3 | Study I only | SAD, MDD-C | Trazadone, Duloxetine, Aripiprazole |
| 4 | Study I only | SAD, MDD-PR | None |
| 5 | Study I only | SAD, MDD-PR | Alprazolam, Zolpidem |
| 6 | Study I only | None | Alprazolam |
| 7 | Study I only | MDD-PR | Clonazepam |
| 8 | Study I only | SAD | None |
| 9 | Study I only | MDD-C | None |
| 10 | Study I only | MDD-PR | Alprazolam |
| 11 | Study I only | SAD, ED-NOS | None |
| 12 | Study I only | None | Citalopram |
| 13 | Study I only | None | None |
| 14 | Study I only | DD | Buspirone |
| 15 | Study I only | None | Venlafaxine, Acamprosate |
| 16 | Active TMS | None | Duloxetine, Clonazepam, Bupropion |
| 17 | Active TMS | SAD | None |
| 18 | Active TMS | SAD, MDD-C | Trazadone, Sertraline, Clonazepam |
| 19 | Active TMS | None | Escitalopram |
| 20 | Active TMS | None | Escitalopram, Trazadone, |
| 21 | Active TMS | MDD-C | None |
| 22 | Active TMS | None | Alprazolam |
| 23 | Active TMS | MDD-PR | Venlafaxine, Alprazolam |
| 24 | Active TMS | SAD, MDD-C | None |
| 25 | Sham TMS | SAD, DD | None |
| 26 | Sham TMS | MDD-PR | None |
| 27 | Sham TMS | None | Clonazepam, Duloxetine |
| 28 | Sham TMS | SAD | Alprazolam, Fluoxetine |
| 29 | Sham TMS | PD | Alprazolam,  Desvenlafaxine |
| 30 | Sham TMS | None | Clonazepam |
| 31 | Sham TMS | MDD-C | Buspirone, Vilazodone, Bupropion, Lorazepam |
| SAD = Social anxiety disorder; DD=Dysthymic disorder; ED-NOS=Eating disorder not otherwise specified; MDD-C=Major depressive disorder, current episode; MDD-PR=MDD, partial remission; PD=Panic disorder | | | |

**Supplementary Figure 1.** ROIs maps**.** X coordinate is provided for each map.


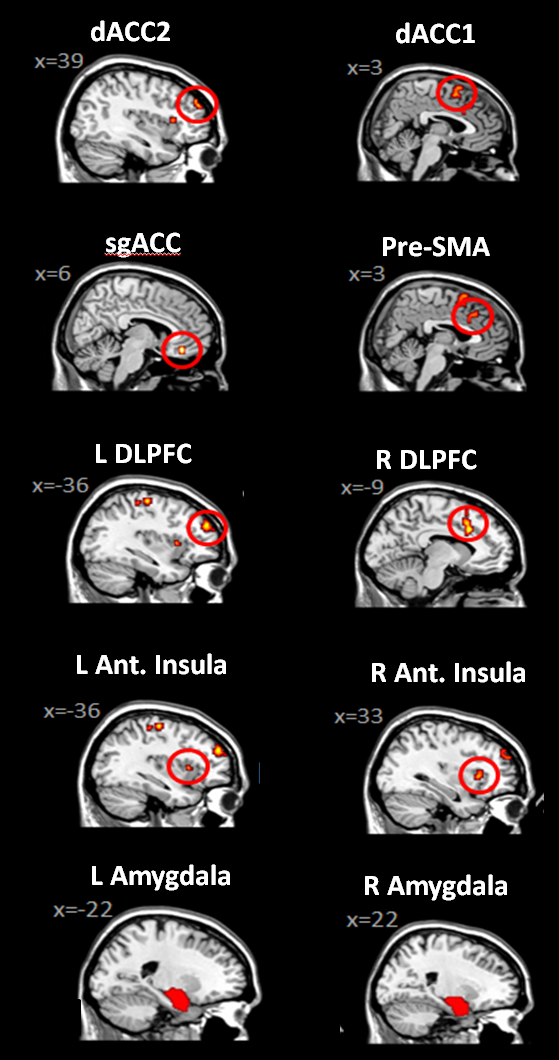


**Supplementary Figure 2.** Functional connectivity (FC) maps for each group (GAD and HC) and task condition (Win and Lose); q_FDR_<0.05. Note that positive FC is indicated by the yellow to red color spectrum and negative FC by cyan to blue spectrum.

**
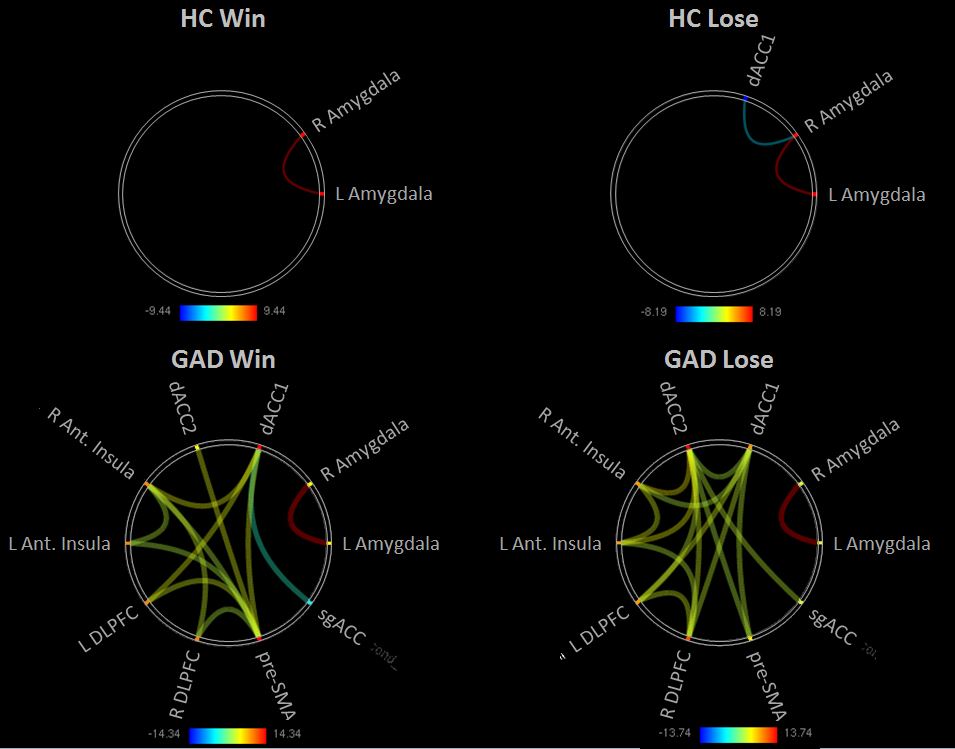
**

**Supplementary Figure 3.** Study II: Bar graphs depicting pre-to-post active vs. sham functional connectivity rTMS effect during the (A) lose and (B) conditions for all tested ROI pairs. Note that the graph for dACC2-sgACC functional connectivity (FC) is identical to the graph presented in Figure 3 (main text) and is presented here again to provide the complete dataset. * p=0.01, ^ p=0.07

**
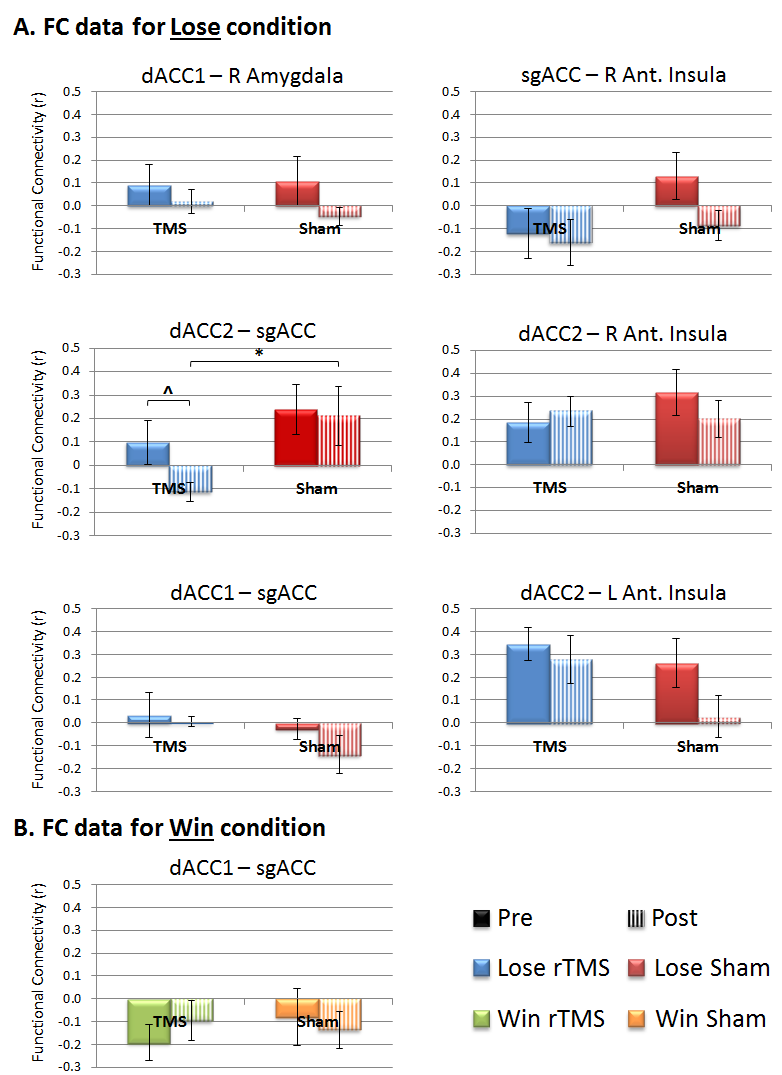
**

**Supplementary Table 2.** Study II: Statistical results for Treatment Condition (Active/Sham rTMS) x Time (Pre/Post Treatment) ANOVA.

| **ROI pair** | **Condition Tested** | **Time**  **Main Effect** | | **Treatment Condition**  **Main Effect** | | **Time X Group**  **Interaction** | |
| --- | --- | --- | --- | --- | --- | --- | --- |
|  |  | F_(1,14)_ | p | F_(1,14)_ | p | F_(1,14)_ | p |
| **dACC1- right Amygdala** | Lose | 1.87 | 0.19 | 0.12 | 0.74 | 0.26 | 0.62 |
| **dACC2-sgACC** | Lose | 2.10 | 0.17 | **5.17** | **0.04** | 1.24 | 0.29 |
| **dACC1-sgACC** | Lose | 0.89 | 0.36 | 2.47 | 0.14 | 0.30 | 0.59 |
|  | Win | 0.05 | 0.82 | 0.14 | 0.71 | 0.69 | 0.42 |
| **sgACC-right AI** | Lose | 1.47 | 0.25 | 2.88 | 0.11 | 0.71 | 0.41 |
| **dACC2-right AI** | Lose | 0.13 | 0.72 | 0.4 | 0.5 | 0.78 | 0.39 |
| **dACC2-left AI** | Lose | 3.22 | 0.09 | 2.53 | 0.13 | 1.03 | 0.33 |
| Abbreviations: AI=Anterior Insula; dACC=Dorsal anterior cingulate cortex; sgACC=Sub-genual ACC. | | | | | | | |
